# Antimicrobial stewardship capacity and antibiotic utilisation practices in the Cape Coast Teaching Hospital, Ghana: A point prevalence survey study

**DOI:** 10.1371/journal.pone.0297626

**Published:** 2024-01-25

**Authors:** Elizabeth Agyare, Joseph Elikem Efui Acolatse, Mavis Puopelle Dakorah, George Akafity, Victoria J. Chalker, Owen B. Spiller, Kristan Alexander Schneider, Saviour Yevutsey, Nana Benyin Aidoo, Sophia Blankson, Frederick Mensah-Acheampong, Robert Incoom, Amanj Kurdi, Brian Godman, Eric Kofi Ngyedu

**Affiliations:** 1 Clinical Microbiology, Cape Coast Teaching Hospital, Cape Coast, Central Region, Ghana; 2 Research and Development Unit, Cape Coast Teaching Hospital, Cape Coast, Central Region, Ghana; 3 Microbiology Department, Bacteriology Unit, Cape Coast Teaching Hospital, Cape Coast, Central Region, Ghana; 4 Clinical Services, National Health Service Blood and Transplant, London, United Kingdom; 5 Medical Microbiology, Division of Infection and Immunity, School of Medicine, Cardiff University, Cardiff, United Kingdom; 6 Department of Mathematics, Hochschule Mittweida University of Applied Sciences, Mittweida, Germany; 7 National Antimicrobial Resistance Secretariat, Office of Pharmaceutical Services, Ministry of Health, Accra, Ghana; 8 Directorate of Nursing Services, Cape Coast Teaching Hospital, Cape Coast, Central Region, Ghana; 9 Directorate of Administrative Services, Cape Coast Teaching Hospital, Cape Coast, Central Region, Ghana; 10 Pharmacy Directorate, Cape Coast Teaching Hospital, Cape Coast, Central Region, Ghana; 11 Strathclyde Institute of Pharmacy and Biomedical Sciences, University of Strathclyde, Glasgow, United Kingdom; 12 Department of Pharmacology and Toxicology, College of Pharmacy, Hawler Medical University, Erbil, Iraq; 13 Centre of Medical and Bio-allied Health Sciences Research, Ajman University, Ajman, United Arab Emirates; 14 Department of Public Health Pharmacy and Management, School of Pharmacy, Sefako Makgatho Health Sciences University, Pretoria, South Africa; 15 Department of Oral and Maxillofacial Surgery, Cape Coast Teaching Hospital, Cape Coast, Central Region, Ghana; University of Nairobi, KENYA

## Abstract

**Introduction:**

Antimicrobial resistance (AMR) is a global threat that necessitates coordinated strategies to improve antibiotic prescribing and reduce AMR. A key activity is ascertaining current prescribing patterns in hospitals to identify targets for quality improvement programmes.

**Methods:**

The World Health Organisation point prevalence survey methodology was used to assess antibiotic prescribing in the Cape Coast Teaching Hospital. All core variables identified by the methodology were recorded.

**Results:**

A total of 78.8% (82/104) patients were prescribed at least one antibiotic, with the majority from adult surgical wards (52.14%). Significantly longer hospital stays were associated with patients who underwent surgery (*p* = 0.0423). “Access” antibiotics dominated total prescriptions (63.8%, 132/207) with ceftriaxone, cefuroxime, and ciprofloxacin being the most prescribed “Watch” antibiotics. The most common indications were for medical prophylaxis (59.8%, 49/82) and surgical prophylaxis (46.3%, 38/82). Over one-third of surgical prophylaxis (34.2%, 13/38) indications extended beyond one day. There was moderate documentation of reasons for antibiotic treatment in patient notes (65.9%, 54/82), and targeted therapy after samples were taken for antimicrobial susceptibility testing (41.7%, 10/24). Guideline compliance was low (25%) where available.

**Conclusions:**

There was high use of antibiotics within the hospital which needs addressing. Identified quality targets include developing surgical prophylaxis guidelines, reviewing “Watch” antibiotic prescribing, and assessing antibiotic durations for patients on two or more antibiotics. Organizational-level deficiencies were also identified that need addressing to help instigate ASPs. These can be addressed by developing local prescribing protocols and antibiotic stewardship policies in this hospital and wider in Ghana and across Africa.

## Introduction

Antimicrobial resistance (AMR) poses significant mortality and healthcare costs with an estimated 1.27 million deaths directly attributed to bacterial AMR in 2019 [1–4]. Global interest and plans to tackle AMR have resulted in initiatives such as the World Health Organization (WHO) Global Action Plan to tackle AMR among others [5–10]. Considering the high prevalence of AMR and infectious diseases across the African continent [1, 11, 12], countries including Ghana have developed and instigated National Action Plans (NAPs) to reduce AMR by encouraging appropriate antimicrobial use across human, animal, and environmental sectors through coordinated activities [13] This One-health approach includes the initiation of antimicrobial stewardship programmes (ASPs) across all sectors of healthcare [9, 14–17], with ASPs described as a coherent set of activities promoting the responsible use of antimicrobials [14, 18, 19]. Though limited resources and trained healthcare personnel in low- and middle-income countries present difficulties in implementing ASPs [20], this is beginning to change with several ASPs successfully introduced across Africa [10, 14, 21–24].

Measuring the quality of antibiotic prescribing patterns via point prevalence surveys (PPS) is a fundamental ASP activity to begin driving improvements [25, 26] such as those concerning prolonged antibiotic use to prevent surgical site infections (SSIs) [27–30]. Other key activities include utilising the WHO Access, Watch, and Reserve (AWaRe) quality indicators, with Access antibiotics favoured as first- and second-line options due to their narrower spectrum and efficacy in a wide range of clinical infections [31, 32]. “Watch” antibiotics are considered to have a higher potential to elicit resistance and should be considered for a limited number of specific infections, whilst “Reserve” antibiotics should only be prescribed for the treatment of multidrug-resistant organisms (MDROs) [31–33]. This is particularly relevant with the current high use of ‘Watch’ and ‘Reserve’ antibiotics in Africa that need to be addressed to curb AMR [34, 35].

Despite challenges since the launch of the Ghanaian NAP in 2017 [13, 36, 37], several PPS studies have been conducted in Ghana in recent years indicating widespread prescribing of antibiotics from the WHO ‘Watch’ list [30, 38–45]. Studies in Ghanaian hospitals have also assessed adherence to antibiotic guidelines in ambulatory care [46] and the burden of SSIs. These studies on SSIs included identifying risk factors, along with concerns about multi-drug resistance and assessing the indications for prescribing ceftriaxone in hospitals [28, 47–50].

Our study builds on these activities by conducting a PPS in the Cape Coast Teaching Hospital (CCTH) as part of a larger ASP to provide baseline data on all core variables of the WHO PPS methodology. The objective was to guide potential quality initiatives that can be undertaken in this and other hospitals in Ghana to improve future antibiotic prescribing. This data, together with cumulative antibiogram data for both in-patients and outpatients, can ultimately be used to develop and refine facility-level antibiotic policies and protocols.

## Materials and methods

### Study site and design

An observational, cross-sectional study following the WHO PPS Methodology was conducted between the 10^th^ and 17^th^ of December 2019 in CCTH [51]. CCTH is a public, 400-bed capacity, tertiary teaching hospital acting as a referral centre to 5.8 million people in the Central, Western, and Western North regions of Ghana [52], with six intensive care unit beds. In 2019, 10,865 patients were admitted amounting to 57,284 patient bed days (CCTH Biostatistics Department, 2019). Hospitalizations were not stratified into acute and high-risk bed occupancies at the time of the survey.

Fourteen wards were included in the study. Wards were classified as being an adult surgical ward (ASW), paediatric medical ward (PMW), adult intensive care unit (AICU), neonatal intensive care unit (NICU), mixed ward (MXW), or an adult medical ward (AMW) per the methodology.

All in-patients at 08:00 am on the day of the survey were included. Exclusion criteria included short-stay patients (not admitted as an inpatient), those discharged before 08:00 am on the day of the survey, and those attending outpatient specialist clinics.

### Data collection and variables

Data were collected by three trained co-investigators based relevant competencies for PPS conduct [53]. The data collection instrument comprised six forms: (i) hospital characteristics data, (ii) a hospital questionnaire, (iii) a ward-level form, (iv) a patient-level form, (v) an indication-level form, and (vi) an antibiotic-level form. All core variables, and two optional variables (guideline compliance and treatment type (empiric or targeted), were recorded. Data for the hospital-level characteristics and hospital questionnaire were obtained from the CCTH antimicrobial stewardship committee and the Biostatistics department. Ward-level data were obtained from each visited ward.

Patient data was accessed via the electronic health system using patients’ identification numbers upon consent. No contact with prescribers was made to clarify the clinical information collected as this could be a focus of future quality improvement programmes. Antibiotic use was stratified by WHO ward classes and indication type (i.e. surgical prophylaxis, SP; medical prophylaxis, MP; community-acquired infections, CAIs; and hospital-acquired infections, HAIs) as defined by the WHO PPS methodology [51]. Only antimicrobials for systemic use were included, these are J01 (antibacterials) and P01A (nitroimidazole derivatives) according to the WHO Anatomical Therapeutic Chemical classification (ATC) [54]. In addition, antibiotics were identified by their WHO AWaRe classification [31, 32]. The durations of surgical prophylaxis were grouped based on administering a single dose multiple doses on one day, and multiple doses over more than one day [51].

Only patients who consented, whether they were prescribed an antimicrobial or not, were used as the denominator for antibiotic prevalence, and patients on antibiotics were used as the numerator.

A pilot including 10 patients from three different wards was conducted for validation of the PPS tool.

### ASP indicators

The WHO questionnaire was used to assess institutional ASP capacity regarding infrastructure, policy and practice, along with monitoring and feedback. This was to enable universal comparison of ASPs in line with other indicators used globally [25, 55, 56].

### Antibiotic use quality indicators

Other indicators included guideline compliance [25, 57–60], treatment type (empiric or targeted) and the route of administration. The Ghanaian NSTGs were used in assessing guideline compliance [44, 61]. The definition of compliance was adapted from Willemsen *et al*. [62]. This was measured based on: (i) the diagnosis of an infection; (ii) whether prophylaxis was indicated; (iii) whether an antibiotic was needed based on the diagnosis; and finally (iv) whether the antibiotic(s) choice was appropriate. The antibiotic with the most recent start date was used for analysis where multiple were prescribed for the same indication. Multiple antibiotics started on the same day for the same indication were considered combination therapy. Full compliance was defined as the right antibiotic choice for an indication of infection or prophylaxis. Partial compliance was defined as when at least one antibiotic was the right choice in the case of combination therapy. Non-compliance was defined as the administration of any antibiotic that was not needed or if antibiotic choice deviated from the NSTG. Duration, dose, and administration route were not included in our compliance definition as these could depend on many clinical factors not recorded in this study.

### Data management and statistical analysis

This was a baseline exploratory study aimed at generating hypotheses, for future quality improvement initiatives considering the few numbers of patients within the dataset. Descriptive and inferential analyses were performed using Microsoft Excel 2016 and R version 4.1.1 respectively. Variables were analysed using one- and two-sided Wilcoxon rank-sum tests, Fisher’s exact tests, Mann-Whitney-U-tests, Kruskal-Wallis tests, and Spearman’s rho tests. A p-value of less than 0.05 was considered statistically significant. No correction for multiple testing was performed due to the study’s exploratory nature.

### Ethics

Ethical approval was granted by the CCTH Research Ethical Review Committee (CCTHERC/EC/2019/075). Patients provided written informed consent before being enrolled in the study. Patients younger than 18 years of age or unconscious at the time of the study had legal guardians provide written informed consent on their behalf.

## Results

### Patient characteristics

A total of 197 in-patients were present at the time of the study, with 195 being eligible. Over half of these (108, 55.4%) provided consent (95% Clopper-Pearson CI: [48.1%, 62.5%]). The observed number of patients was within the expected range during the study period (expected number of visits 208.37; 95% CI: [170.67,225.33]), reflecting that the observed week was representative. Consent to participate was not equally distributed across hospital wards (S1 Table). Of the study participants, males comprised 38%. Four patients had no prescribing information available; consequently, 104 patients were used as the denominator for analysis. The full demographics and antibiotic use by WHO ward classifications are summarized in Tables 1 and 2.

**Table 1:** Demographics of 108 patients and antibiotics.

DEMOGRAPHICS	ANTIBIOTICS (%)	NO ANTIBIOTICS (%)	INFORMATION NOT AVAILABLE (%)	TOTAL (%)
Sex Male	33	8	0	41
Sex Female	49	13	4	66
Sex Unknown	0	1	0	1
**Total**	82	22	4	108
Age neonates <28 days	6	1	3	10
Age 2 months to 12 years	17	1	1	19
Age 13–17 years	4	3	0	7
Age ≥18 years	55	17	0	72
**Total**	82	22	4	108

**Table 2:** Antibiotic use by ward.

WHO ward classification	Paediatric medical ward	Neonatal ICU	Adult ICU	Adult medical ward			Mixed ward		Adult surgical ward ASW			Total
CCTH Ward	PED	NICU	ICU	FMW	MMW	AEU	EW	ET	SWM	FSW	OG	All
Patients surveyed	25	10	4	5	2	5	1	1	16	17	22	108
Patients on antibiotics (%)	21 (84%)	6 (60%)	4 (100%)	2 (40%)	2 (100%)	2 (40%)	1 (100%)	0 (0%)	13 (81.3%)	13 (76.5%)	18 (81.8%)	82* (78.8%)
One antibiotic	3	0	0	1	0	0	1	0	0	4	3	12
Two antibiotics	6	3	2	1	2	1	0	0	5	4	13	37
Three antibiotics	7	0	1	0	0	1	0	0	6	3	2	20
Four antibiotics	3	2	0	0	0	0	0	0	1	1	0	7
> Four antibiotics	2	1	1	0	0	0	0	0	1	1	0	6

Table 2. CCTH Ward abbreviations: PED–paediatric ward, NICU—neonatal intensive care unit, ICU—intensive care unit, FMW—female medical ward, MMW—male medical ward, AEU—accident and emergency unit, EW—executive ward, ET—emergency triage assessment & treatment unit, SWM—male surgical ward, FSW—female surgical ward, OG—obstetric and gynaecological ward.

*Denominator was 104 since 4 patients had no prescribing data available

The median age of surveyed male patients (27 years; IQR: 12.00–50.00) and that of females (30 years; IQR: 17.25–51.75) was not significantly different (2-sided Mann-Whitney-U-test: P = 0.5). The mean age in the adult wards also did not differ (Kruskal-Wallis test: P = 0.641).

The length of hospital stays until the point of the survey did not differ between males and females (2-sided Mann-Whitney-U-test, P = 0.169). The length of stay till the survey date was shorter in the mixed wards (MXW) and had the largest range in the adult medical wards (AMW). However, the overall length of hospital stays was not different between the wards; Kruskal-Wallis test: P = 0.107). No correlation between age and the length of hospital stays was found (Spearman’s rho = -0.164, two-sided test for Spearman’s rho: P = 0.0958). As expected, patients undergoing surgery had longer hospital stays (1-sided Wilcoxon rank-sum test: P = 0.042). No association between gender and surgical admission was found (Fisher’s exact test, P = 1). As the length of stay was not measured until discharge, it is possible patients may have received more antibiotics after the survey date.

Data on study variables and antibiotic use was available for 104 patients. Three patients (2.9%, 3/104), had a central venous catheter, with two of them from the AICU and the last from the AMW. Additionally, four patients (3.8%, 4/104) were found to be intubated (three from the AICU and one from the AMW). Twenty-one (20.2%, 21/104) patients had a peripheral catheter with 16 (76.1%) of them on antibiotics. Thirteen patients (12.5%, 13/104) had a urinary catheter out of which 10 (76.9%) were on antibiotics. Both peripheral and urinary catheters had a majority of patients coming from the AICU and adult surgical wards (ASW).

### Prevalence of antibiotic use

The prevalence of patients on antibiotics was 78.8% (82/104, 95% Clopper-Pearson CI: [69.7%, 86.2%]). The prevalence of antibiotic use did not differ between sex of patients surveyed (OR = 1.09, 95% CI: [0.37,3.40]; Fisher’s exact test: P = 1). The age of patients with or without antibiotic treatment also did not differ (two-sided Mann-Whitney-U test: P = 0.386). The length of hospitalisation was significantly longer among patients on antibiotics (one-sided Mann-Whitney-U test, P = 0.0002). The highest prevalence of antibiotic use among patients was found in AICU (100%; 4/4), followed by the paediatric ward (PMW) (87.5%; 21/24), NICU (85.7%; 6/7), and the ASW (80%; 44/55). In the AMW the prevalence of antibiotic use was 57.1% (4/7), while in the MXW it was 42.9% (3/7).

Of 82 patients on antibiotics, 53.6% were in the ASW followed by the paediatric ward (PMW) (25.6%), whereas the AICU contributed the least. Adjusted percentages reflecting the relative size of the medical wards and hence the relevance of the wards to the overall number of patients on antibiotics are detailed in S1 Table. This adjustment demonstrates that whilst the AICU had the highest prevalence of antibiotic use it is a relatively small ward. Consequently, contributed the least to the overall number of patients prescribed antibiotics (S1 Table).

### Number of antibiotics prescriptions

Patients who were treated with antibiotics received an average of 2.5 antibiotics (207/82; SD 1.19). Whilst males and females were equally likely to be treated with antibiotics, males received a significantly higher number of antibiotics (2.82 vs. 2.32; one-sided Mann-Whitney-U test; P = 0.002). No correlation was found between the number of antibiotics and age (Spearman’s rho = -0.164; two-sided test for Spearman’s rho P = 0.096). Not surprisingly, the number of antibiotics correlated with the length of the hospital stay (Spearman’s rho = 0.4804619, two-sided test for Spearman’s rho P = 2.447e-07). The number of antibiotics prescribed differed significantly across the hospital wards (Kruskal-Wallis test: P = 0.0146), with the highest average number in the AICU (3.5 antibiotics per patient), and the lowest in the MXW (0.9 antibiotics per patient).

Overall, the ASW accounted for the largest percentage of antibiotics used (49.27%) followed by the paediatric medical ward (PMW) (25.35%) after correcting for included patients out of the total eligible present in the ward. The PMW, NICU, and AICU wards contributed higher percentages to the number of antibiotics prescribed per patient than all other wards. This indicates that, on average, more antibiotics are used per patient in these wards than in the others (S1 Table).

### Types of antibiotics

**Fig 1:**
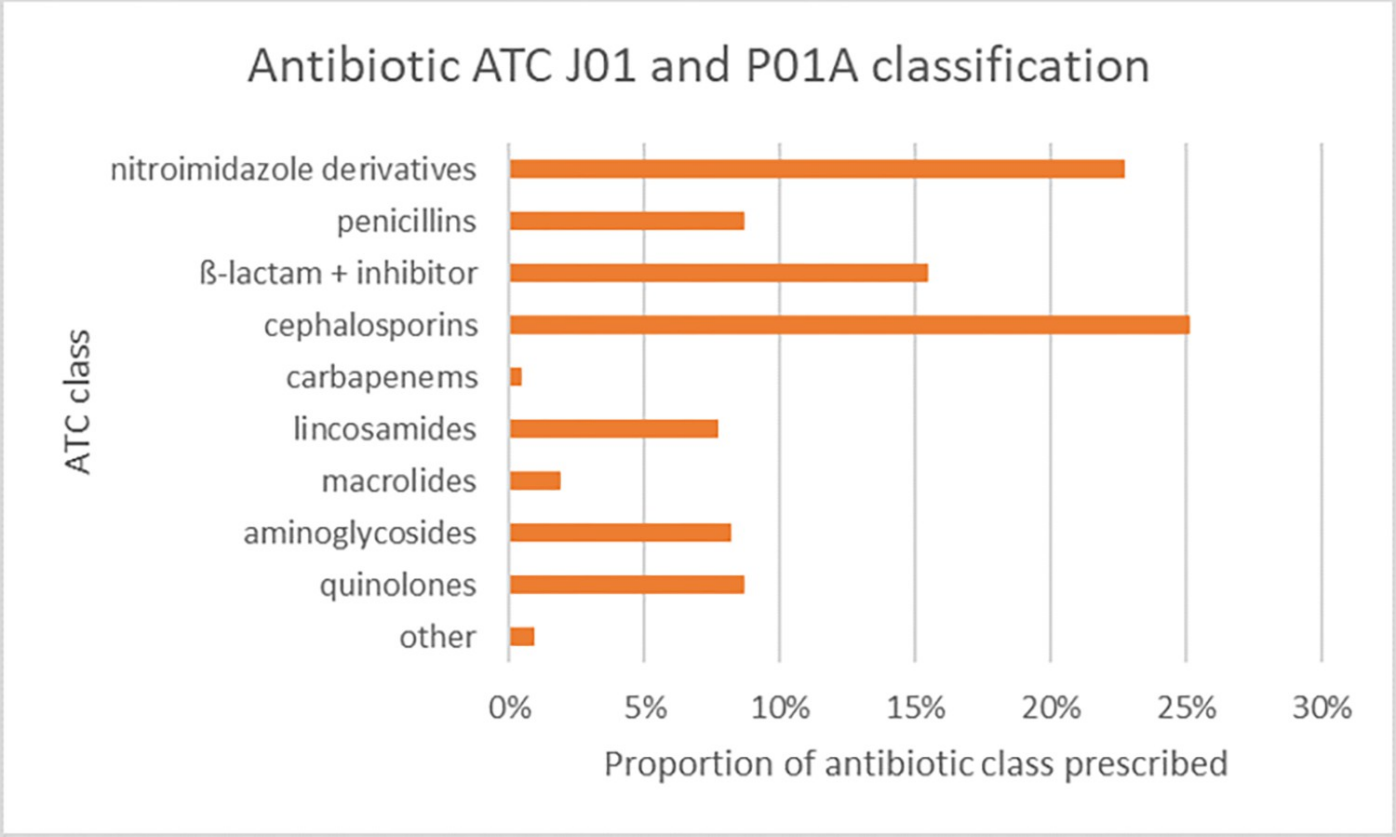
Proportion of antibiotic class prescribed: Antibacterials for systemic use (J01) and nitroimidazole derivatives (P01A) Anatomical Therapeutic Classification (ATC).

Fifteen different types of antibiotics were used, with a total of 207 antibiotics prescribed to patients over their entire course of admission. The five most commonly prescribed antibiotics were metronidazole (22.7%) and amoxicillin-clavulanic acid (15.5%) which are both “Access” antibiotics, followed by ceftriaxone, cefuroxime, and ciprofloxacin which are “Watch” antibiotics. According to ATC classifications, nitroimidazole derivatives, ß-lactam + inhibitor, and 2^nd^/3^rd^ generation cephalosporins amounted to 64.3% of all antibiotics prescribed, with cephalosporins comprising the majority (Fig 1). Parenteral delivery accounted for over 77% of all prescriptions compared to oral treatments (Table 3).

**Table 3:** Antibiotic prescribing proportion by AWaRe category, route of administering, and ward classifications in CCTH.

Antibiotic	AWaRe category	Total (%)		Parenteral antibiotics (%)	Oral antibiotics	Paediatric medical ward	Neonatal ICU	Adult ICU	Adult medical ward		Mixed ward			Adult surgical ward ASW		
						PED	NICU	ICU	MMW	FMW	AEU	EW	ET	FSW	OG	SWM
**metronidazole**	Access	47(22.7)	37(23.1)		10(21.8)	10(16.9)	1(5.3)	2(14.3)	0(0)	0(0)	1(20)	0(0)	0(0)	10(33.3)	14(40)	9(24.3)
**amoxicillin clavulanic acid**	Access	32(15.5)	23(14.4)		9(19.1)	6(10.2)	0(0)	2(14.3)	1(25)	1(33.3)	1(20)	0(0)	0(0)	3(10)	13(37.1)	5(13.5)
**benzylpenicillin**	Access	8(3.9)	8(5)		0(0)	2(3.4)	6(31.6)	0(0)	0(0)	0(0)	0(0)	0(0)	0(0)	0(0)	0(0)	0(0)
**phenoxymethyl penicillin**	Access	3(1.5)	2(1.3)		1(2.1)	2(3.4)	0(0)	0(0)	0(0)	0(0)	0(0)	0(0)	0(0)	0(0)	1(2.9)	0(0)
**flucloxacillin**	Access	7(3.4)	5(3.1)		2(4.3)	2(3.4)	3(15.9)	0(0)	1(25)	1(33.3)	0(0)	0(0)	0(0)	0(0)	0(0)	0(0)
**clindamycin**	Access	16(7.7)	11(6.9)		5(10.6)	8(13.6)	0(0)	2(14.3)	0(0)	0(0)	0(0)	0(0)	0(0)	2(6.7)	1(2.9)	3(8.1)
**amikacin**	Access	4(1.9)	4(2.5)		0(0)	0(0)	0(0)	0(0)	0(0)	0(0)	0(0)	0(0)	0(0)	0(0)	0(0)	4(10.8)
**gentamicin**	Access	13(6.3)	13(8.1)		0(0)	5(8.5)	6(31.6)	1(7.1)	0(0)	0(0)	0(0)	0(0)	0(0)	0(0)	1(2.9)	0(0)
**trimethoprim-sulfamethoxazole**	Access	2(1.0)	0(0)		2(4.3)	2(3.4)	0(0)	0(0)	0(0)	0(0)	0(0)	0(0)	0(0)	0(0)	0(0)	0(0)
**ceftriaxone**	Watch	25(12.1)	24(15)		1(2.1)	9(15.3)	0(0)	3(21.4)	0(0)	0(0)	1(20)	0(0)	0(0)	5(16.7)	3(8.6)	4(10.8)
**cefuroxime**	Watch	24(11.6)	17(10.6)		7(14.9)	9(15.3)	0(0)	0(0)	0(0)	0(0)	0(0)	1(100)	0(0)	5(16.7)	2(5.7)	7(18.9)
**cefotaxime**	Watch	3(1.4)	3(1.9)		0(0)	0(0)	3(15.9)	0(0)	0(0)	0(0)	0(0)	0(0)	0(0)	0(0)	0(0)	0(0)
**meropenem**	Watch	1(0.5)	1(0.6)		0(0)	0(0)	0(0)	1(7.1)	0(0)	0(0)	0(0)	0(0)	0(0)	0(0)	0(0)	0(0)
**azithromycin**	Watch	4(1.9)	1(0.6)		3(6.4)	1(1.7)	0(0)	1(7.1)	1(25)	0(0)	1(20)	0(0)	0(0)	0(0)	0(0)	0(0)
**ciprofloxacin**	Watch	18(8.7)	11(6.9)		7(14.9)	3(5.1)	0(0)	2(14.3)	1(25)	1(33.3)	1(20)	0(0)	0(0)	5(16.7)	0(0)	5(13.5)
**Total**		**207**		160	47	59	19	14	4	3	5	1	0	30	35	37

Table 3. CCTH Ward abbreviations: PED–paediatric ward, NICU—neonatal intensive care unit, ICU—intensive care unit, FMW—female medical ward, MMW—male medical ward, AEU—accident and emergency unit, EW—executive ward, ET—emergency triage assessment & treatment unit, SWM—male surgical ward, FSW—female surgical ward, OG—obstetric and gynaecological ward.

There was no difference in the types of antibiotics used in males and females (Fisher’s exact test with simulated p-values based on 500,000 Monte Carlo replicates: P = 0.692). The age of patients differed significantly across the different antibiotic treatments (Kruskal-Wallis test: P = 3.59e-09). This is hardly surprising because certain antibiotics were prescribed mainly in the PMW and NICU. The length of stay was different across the different antibiotic treatments (Kruskal-Wallis test: P = 0.004), which may be due to specific antibiotics being given for surgical prophylaxis as patients with surgery had longer hospital stays, with the same situation observed in the AICU.

The type of antibiotics prescribed differed substantially across WHO ward classifications. The largest variety of antibiotics was used in the PMW (12 different antibiotics), followed by the ASW (9 different antibiotics). In the NICU and AMW, only six and four different antibiotics were used respectively. Four antibiotics (benzylpenicillin, cefotaxime, phenoxymethylpenicillin, and trimethoprim-sulfamethoxazole) were almost exclusively prescribed in the PMW and NICU. Amikacin was only prescribed in the ASW and meropenem in the AICU. Three antibiotics from the ‘Watch’ list were frequently used: ceftriaxone, cefuroxime, and ciprofloxacin. Ceftriaxone, cefuroxime, and ciprofloxacin each amounted to 16.7% of the antibiotics used in the MXW. In the AMW, ciprofloxacin amounted to 28.6% of the antibiotics prescribed. Ceftriaxone and cefuroxime were commonly used in the ASW, together amounting to 25.5% of the antibiotics used in this ward.

### Antibiotic prescribing quality indicators

Thirty-eight patients had surgery since admission. The majority (60.5%) received multiple prophylactic doses on one day (SP2). The top three sites for surgical prophylaxis were obstetrics and gynaecologic surgery (13/38); those related to skin, soft tissue, bone and joint surgery (9/38), and gastrointestinal tract surgeries (7/38).

In some patients, antibiotics were prescribed for multiple indications resulting in 110 indications for 82 patients. Thus, 14 patients were treated for CAI (14/82, 17.1%), nine for HAI (9/82, 11.0%), 38 (38/82, 46.3%) for SP, and 49 (49/82, 59.8%) treated as MP, where MP includes the use of antibiotics to prevent infections in cases such as open fractures or wounds not instigated for the purposes of a surgery. Concerning the nine patients with HAIs, two had multiple HAI indications. A total of 27 CAIs and HAIs were diagnosed. The most common diagnoses for these two indications were: pneumonia (PNEU) (7/27); cellulitis, wound, deep soft tissue not involving bone, not related to surgery (SST-O) (6/27); clinical sepsis i.e., suspected bloodstream infection without lab confirmation (CSEP) (5/27); and surgical site infections (SST-SSI) (3/27). Gastrointestinal infections (GI) and septic arthritis/osteomyelitis (BJ-O) had equal frequencies (2/27).

Fourteen patients (60.8%, 14/23) diagnosed with either a CAI or HAI had no samples taken for antimicrobial susceptibility testing (AST) for suspected infections during the study. For the nine remaining patients (39.1%, 9/23), samples were taken for AST for targeted therapy. An additional sample was taken from one patient undergoing MP. As a result, samples were taken from 10 patients in total. A total of 13 samples from varying body sites (blood, urine, wound, sterile fluid, sputum, and others) were taken i.e. multiple samples were taken from some patients. Five samples were positive for microbial growth while five were negative and three results were unavailable at the time of the survey. This resulted in 10 patients (41.7%, 10/24) receiving AST results acting as a basis for targeted therapy. The only resistant isolate recorded was an *Escherichia coli* isolate resistant to 3^rd^-generation cephalosporins. Other identified isolates included a methicillin-susceptible *Staphylococcus aureus*, *Pseudomonas aeruginosa*, *Proteus mirabilis*, and coagulase-negative *Staphylococci* spp.

Guideline compliance was available for 53.7% of patients on antibiotics. The remaining 38 patients on antibiotics had treatment indications for conditions with no guidelines available and could not be assessed. Full guideline compliance was 25%, whereas partial compliance stood at 43.2%. Non-compliance was also found to be 31.8% among treated patients.

Overall, antibiotics from the ‘Access’ list dominated total antibiotic use. However, three of the five most commonly prescribed antibiotics were from the ‘Watch’ list. There was limited use of carbapenems and no antibiotic from the ‘Reserve’ list was recorded. All quality indicators measured during the study are outlined in Table 4.

**Table 4:** Quality indicators.

Quality indicator	Number	% proportion
**Reason for antibiotic in notes**	54/82	65.9%
**Guideline compliance**	11/44	25%
**Patients with results for samples taken for AST**	10/24	41.7%
**Duration of Surgical Prophylaxis**		
Surgical Prophylaxis one dose	2/38	5.3%
Surgical Prophylaxis multiple doses on one day	23/38	60.5%
Surgical Prophylaxis multiple doses for more than one day	13/38	34.2%
**Antibiotic prevalence by AWaRe Classification**		
Access	132/207	63.8%
Watch	75/207	36.2%
Reserve	0/207	0%

Table 4. AST–Antimicrobial Susceptibility Testing, AWaRE–Access, Watch, and Reserve antibiotic classification

### ASP structural indicators

Data from the questionnaire showed that CCTH had a moderate capacity for implementing an ASP by meeting 14 out of 34 indicators. The majority of met indicators were under infrastructure. However, CCTH has deficits concerning policy and practice and monitoring and evaluation. These two categories also contained the majority of the process indicators with the results summarized in Table 5 and full details of the questionnaire in S2 Table.

**Table 5:** Summary of CCTH performance regarding hospital questionnaire indicators for assessing ASP capacity.

Indicator category	Number of indicators within each category	Number of indicators met
Infrastructure	14	12
Policy and Practice	8	1
Monitoring and Feedback	12	1

## Discussion

To the best of our knowledge, this is one of the first studies in Ghana which systematically reports on the core variables of the WHO PPS standard. Moreover, to facilitate comparison and reproducibility we clearly defined the method for assessing the optional variable on guideline compliance.

The prevalence of prescribing at least one antibiotic in CCTH was 78.8%. Within Ghana, this prevalence was higher than in other tertiary hospitals across the country. One study also using the WHO methodology reported a prevalence of 67.3% for only one facility classified as a tertiary hospital among the participating sites [42]. Further studies exhibited similar prevalence rates of 65.6% (95% CI: [60.4,70.6]) among a paediatric population from two tertiary hospitals [45] and 66.4% to 47.8% among seven hospitals in a multicentre study [41]. Labi *et al*. (2018) reported a lower prevalence of antibiotic use in tertiary hospitals, namely 51.4% [63], whereas Ankrah *et al*. reported a prevalence of 53.3% [40]. Finally, a prevalence of 54.9% and 66.7% at two different time points in the same tertiary hospital were recorded by Dodoo *et al*. [39].

Compared to secondary-level hospitals in the country, the prevalence of antibiotic use in CCTH was higher than that of the Police hospital (65.0%) but lower than that of the Keta Municipal hospital (82.0%) [44].

In comparison to other African countries, the prevalence of antibiotic prescribing in CCTH was higher than seen among hospitals in Tanzania (44% - 62.3%) [64, 65], Kenya (46.7% - 66.7%) [66–68], South Africa (33.6% - 44%) [69, 70] and Zambia (59%) [71]. It was, however, similar to Uganda (74%) [60] and Botswana at 70.6% [72]; but lower than reported in Nigeria (80.6%) and Eswatini (88.2%) [73, 74].

When assessing the quality of prescribing using the AWaRe classification, a high proportion of “Access” antibiotic use (63.8%) was found, similar to other Ghanaian hospitals. Labi et al. (2018) reported 62.2% of prescriptions from the “Access” list, with this same category amounting to over 60% and 66% of prescriptions in the studies of Ankrah *et al*. and Amponsah *et al*., respectively [40, 42, 63]. There was relatively low use of “Watch” antibiotics (36.2%) and no use of “Reserve” antibiotics on the day of the survey in our study. This was an encouraging observation, as “Watch” and “Reserve” antibiotics are identified as good targets for ASPs as there have been concerns with the extent of prescribing of ‘Watch’ and ‘Reserve’ antibiotics across sectors in Africa [33, 34, 71, 75].

Overall, these results indicate a current high prevalence of antibiotic use in CCTH. Consequently, prompt attention through ASP interventions is needed to address any inappropriate prescribing especially among ‘Watch’ antibiotics [14, 17, 34]. The reasons for this could be attributed to a statistically significant majority of antibiotic use emanating from ASW, coupled with the high number of patients on surgical and medical prophylaxis, and significantly longer hospital stays for patients who underwent surgery up until the survey date.

Whilst only 5.3% of patients undergoing surgery received a single dose pre-operatively, 60.5% received multiple doses on the first day, with the remainder (34.2%) receiving extended prophylaxis for >1 day. Though this can be improved given the negative implications of extended prophylaxis [29], it is encouraging compared to other studies in Ghana. The overall duration of prophylaxis in a secondary level was 6.9 +/- 2.1 days [28] with two other hospitals combining to record over 70% of surgical prophylaxis lasting over one day [44]. Additionally, extended surgical prophylaxis comprised 88.4% of cases in a PPS enrolling 540 surgical patients from 10 systematically sampled Ghanaian hospitals where almost half of the included patients were from tertiary hospitals [30]. Another teaching hospital recorded 69.6% of patients receiving surgical prophylaxis for more than one day [76], with this figure increasing to 74.7% in a multicentre study with four teaching hospitals included [41]. Having said this, the prevalence of extended prophylaxis observed is appreciably lower than seen in a number of other African countries. For instance, in Botswana extended prophylaxis (> 1 day) was common and greatest among primary and tertiary hospitals (100% of patients surveyed), similar to a study conducted in Nigeria where all antibiotics prescribed to prevent SSIs were administered for > 1 day [73, 77]. In one study conducted in Kenya, 76.9% of surveyed patients received antibiotics to prevent SSIs for > 1 day [68]. Tanzania and Zambia also recorded antibiotics prescribed for > 1 day to prevent SSIs for 97% and 96.5% of patients respectively (39). This was identical to Uganda where 98.4% of patients had multiple doses of antibiotics for > 1 day to prevent SSIs in surveyed hospitals [60].

Whilst encouragingly, over 68% of all prescribed antibiotics were from the “Access” group of antibiotics, cefuroxime and ceftriaxone were identified as the most commonly prescribed “Watch” antibiotics in the top five prescriptions, which was similar to other PPS studies conducted among hospitals in Ghana [30, 40–42, 63]. These other PPS studies also had respiratory infections as one of the leading diagnoses of CAI or HAI among surveyed patients. Whilst this may immediately suggest inappropriate prescribing practices, the NSTGs recommend cefuroxime as a second-line treatment for pneumonia and ceftriaxone as second-line therapy for hospitalized patients with a severity score of >2 [61]. The Ghanaian STG also indicates ceftriaxone as the first-line choice for aspiration pneumonia. Consequently, four out of seven patients with this diagnosis received at least one of these antibiotics. Aside from a majority of pneumonia cases in the PMW (42.9%, 3/7), this ward also admitted all patients below 18 years of age undergoing surgical procedures. This contributed to recording the highest use of “Watch” antibiotics (n = 18) in this ward. The second leading contributor to the prescribing of “Watch” antibiotics came from the ASW where antibiotic prescribing guidance in the NSTGs is limited for many of the procedures performed. These highlight systematic problems hindering ASP implementation in Ghana as well as the paucity of well-defined definitions for measuring guideline adherence among other PPS studies. This will require in-depth exploration, including a review and adaptation of the NSTGs to the local context, which we have started. The high proportion of parenteral to oral antibiotics also showed opportunities for oral switching which could lead to reduced time in the hospital among other benefits [9, 78, 79].

We believe this study was the first to measure and fully report ASP capacity indicators in Ghana to enable robust comparisons among different institutions and identify organisational-level areas in need of intervention. Regarding infrastructure, a lack of salary support for dedicated antimicrobial stewardship time and a lack of an outpatient parenteral antibiotic therapy (OPAT) unit were the only two unmet indicators. Salary support is crucial to sustained ASP interventions as it has been estimated that a 0.87 full-time equivalent (FTE) translating to 393 to 2680 hours annually are needed for a single ASP intervention, especially in the beginning stages [80]. Grant stipends used to cater for salary support for investigators during the project were as high as 83% of their FTE. Consequently, a more sustainable funding approach is necessary going forward. Having said this, we are already seeing multiple ASPs being performed across Africa in recent years, and this is likely to grow given the increasing need to reduce AMR as part of NAPs [12, 14, 23, 24].

Monitoring and feedback, together with Policy and practice, performed poorly with only one indicator met in each category. The lack of local guidelines coupled with the absence of susceptibility data to guide prescribing was a key finding in this study. This subsequently led to the development of a cumulative antibiogram as a baseline to begin the adaption and development of local guidelines where these were absent. Prior to this study, there was no monitoring of whether prescribers recorded indications for all antibiotic prescriptions this hampered data collection and analysis procedures as the reason for prescribing had to be elicited from lengthy notes in some cases. This could additionally serve as a problem for reimbursement from the National Health Insurance as a clear diagnosis and indication is required. Yearly PPS studies were instituted by the CCTH Drugs and Therapeutics Committee (DTC) to institutionalise the monitoring of this indicator as a continuous quality improvement programme. An antibiotics protocol mandating the recording of indications was developed and delivered to prescribers together with PPS and cumulative antibiogram findings in 2021 to reinforce this with these results being part of a larger ASP to be published elsewhere.

Due to the hospital’s ethics regulations, our study required written informed consent from patients. This is different from other PPS studies conducted in Ghana, which typically waived informed consent [30, 39, 41, 45] or deemed consent not applicable [38, 40, 44, 63]. Consequently, as a limitation, informed consent could only be obtained from 57% of patients. Informed consent was typically not given because patients were unconscious or the parents/legal guardians were absent due to various reasons when investigators approached them (the hospital provides total nursing care and does not require the presence of accompanying visitors). Notably, only a handful of patients denied informed consent. While the experiences from this study were evaluated leading to the DTC institutionalising this activity as mentioned previously thereby removing the requirement for consent, the nature of failure to obtain informed consent can be considered random, so that only the sample size of our PPS was reduced, but no bias introduced.

Comparison of the prevalence between CCTH and other hospitals should be done carefully as some studies included multiple sites with varying bed capacities, patient numbers, and different data collection. The heterogeneity in hospital types (tertiary vs secondary) and the time of year the studies were conducted may also present key confounders in direct comparisons. Despite these reasons, we believe our comparisons are reasonable regarding the prevalence estimate and confidence intervals (95% Clopper-Pearson CI: [69.7%, 86.2%]) found in this study.

However, the main purpose of this study was exploratory and aimed at identifying quality improvement targets as part of an ASP with these findings later corroborated with subsequent PPS conducted in CCTH over the years with higher enrolment numbers.

Samples taken for AST were 43.5%. This was rather encouraging, especially when compared to other studies in Ghana which measured from 1.2% to 8% [30, 39–42, 44]. Of particular interest was an *Escherichia coli* isolate with resistance to third-generation cephalosporins. This *E*. *coli* isolate was from an ICU patient diagnosed with HAI causing suspected sepsis and subsequent culturing of cerebrospinal fluid. The prospective creation of a year-long cumulative antibiogram in 2020 [81], coupled with an environmental surveillance of inpatient wards for extended-spectrum beta-lactamase (ESBL) and carbapenemase-producing Gram-negative bacteria in 2021 [82], indicated high prevalence rates of ESBL *E*. *coli* among both patients and CCTH ward environments. This emphasizes the importance of cognisance of antibiograms for empiric prescribing in CCTH. Another patient had a positive culture for *P*. *aeruginosa*, highlighting the need to improve documentation of indications by clinicians as this could affect requests for AST. Sample taking for AST is crucial for directing definitive therapy and even improving empiric therapy through the generation of cumulative antibiograms, and we will be looking to improve the taking of ASTs in the future. Unfortunately, no analysis was conducted to determine if the initial antibiotics prescribed were changed with the availability of AST findings; however, they will also be a future target for quality improvement studies and programmes.

Guideline compliance was low at 25%. This is a concern as poor compliance impacts subsequent patient care [57]. Regular updates are needed as well as incorporating infections where guidance is insufficient or lacking to improve their acceptance and use [57, 58]. Overall, we were unable to assess compliance in 46.3% of situations as there was no current guidance, especially for many surgical procedures. The use of mobile applications could enhance the uptake of these guidelines by prescribers building on initiatives in other African countries, and we will be following this up [38].

Future studies will also include developing a regression model to determine predictors of antibiotic use with a larger dataset. This will seek to reduce artefacts and fine-tune stewardship approaches based on identified targets of high priority.

## Limitations

There was a low percentage of consent provided by patients eligible on the survey date which may limit the comparability of our results to other studies. This study also did not record optional variables regarding comorbidities such as malaria, HIV, or tuberculosis infections among surveyed patients which may have influenced the extent of antibiotic prescribing. There was also missing data. However, this is normal for PPS studies, highlighting potential areas for future quality improvement programmes. Despite these limitations, we believe our findings are robust and already providing guidance for future quality improvement programmes in the hospital.

## Conclusions

Inappropriate antibiotic use was attributed to adult surgical wards, increased length of hospitalisation up to the day of the survey, and admission to the adult ICU. These generated hypotheses will be examined in consequent PPS in CCTH and could be explored in other hospitals to determine sources contributing to inappropriate antibiotic use in those settings.

At the patient level, updating of NSTGs on surgical prophylaxis, increasing the utilisation of AST for suspected CAI/HAIs, and reviewing prescribing indications for patients on “Watch” antibiotics required improvement. Patients given more than two antibiotics during their hospitalization were also identified as areas in need of quality improvement. These will be the focus of the CCTH ASP in subsequent years.

Major organizational deficiencies regarding policy and practice indicators, as well as those for monitoring and feedback were identified within CCTH. This led to initiatives which include the development of a local prescribing protocol guided by a facility-specific cumulative antibiogram and an antibiotic stewardship policy instituting the use of repeated PPS in CCTH. These helped to address many indicators which were previously lacking in the institution. These can act as viable starting points for other hospitals with inadequate ASP capacity in similar contexts within Africa.

## Supporting information

S1 Table Adjusted proportions of included patients out of total eligible patients on antibiotics number and use. AICU—Adult Intensive Care Unit, AMW—Adult Medical Ward, ASW—Adult Surgical Ward, MXW—Mixed Ward, NICU—Neonatal. Intensive Care Unit, and PMW—Paediatric Medical Ward.
(PDF)

S2 Table Hospital questionnaire on structural and process indicators for assessing ASP capacity in CCTH [52]. (PDF)
