# Supplementary material for: Antimicrobial stewardship capacity and antibiotic utilisation practices in the Cape Coast Teaching Hospital, Ghana: A point prevalence survey study
Source: PLoS One. 2024 Jan 25;19(1):e0297626. doi: 10.1371/journal.pone.0297626 (PMC10810544; doi:10.1371/journal.pone.0297626)
Supplement: S1 Table — AICU—Adult Intensive Care Unit, AMW—Adult Medical Ward, ASW—Adult Surgical Ward, MXW—Mixed Ward, NICU—Neonatal. Intensive Care Unit, and PMW—Paediatric Medical Ward. (PDF) [file pone.0297626.s001.pdf]

Supplementary Data

S1 Table. Adjusted proportions of included patients out of total eligible patients on antibiotics number and use.

**Ward**	Number of eligible patients	Number of patients with consent	Proportion of consenting patients out of all eligible (%)	Proportion of patients on antibiotics (%)	Weighted correction of total patients with antibiotic prescriptions (%)	total number of antibiotics used (%)	Weighted correction of total number of antibiotics used (%)
**AICU**	4	4	100	4.88	2.77	6.76	3.96
**AMW**	26	7	26.9	4.88	10.30	3.38	7.35
**ASW**	94	55	58.5	53.66	52.14	49.28	49.27
**MXW**	16	7	43.8	3.66	4.75	2.9	3.88
**NICU**	19	10	52.6	7.32	7.90	9.18	10.2
**PMW**	38	25.0	65.8	25.6	22.1	28.5	25.4

AICU - Adult Intensive Care Unit, **AMW** - Adult Medical Ward, **ASW** - Adult Surgical Ward, **MXW** - Mixed Ward, **NICU** - Neonatal Intensive Care Unit, and **PMW** - Paediatric Medical Ward.
