# Supplementary material for: Antimicrobial stewardship capacity and antibiotic utilisation practices in the Cape Coast Teaching Hospital, Ghana: A point prevalence survey study
Source: PLoS One. 2024 Jan 25;19(1):e0297626. doi: 10.1371/journal.pone.0297626 (PMC10810544; doi:10.1371/journal.pone.0297626)
Supplement: S2 Table — (PDF) [file pone.0297626.s002.pdf]

S2 Table. Hospital questionnaire on structural and process indicators for assessing ASP capacity in CCTH [52].

	Question	Answer
**Code**	**Infrastructure**	2019
**I1**	Does your facility have a functional Drugs and Therapeutics Committee in the hospital?	yes
**I2**	Does your facility have a functioning Infection Prevention & Control Committee in the hospital?	yes
**I3**	Does your facility have a functioning Committee on pharmacovigilance in the hospital?	yes
**I4**	Does your facility have a microbiological laboratory/division within the facility?	yes
**I5**	Does your facility have access to microbiological services outside the hospital?	yes
**I6**	Does your facility have a formal antimicrobial stewardship programme accountable for ensuring appropriate antibiotic use?	yes
**I7**	Does your facility have a formal organisational structure responsible for antimicrobial stewardship? (eg, a multidisciplinary committee focused on appropriate antibiotic use, pharmacy committee, patient safety committee, or other relevant structure)	yes
**I8**	Is an antimicrobial stewardship team available at your facility? (eg, greater than one staff member supporting clinical decisions and implementing a comprehensive programme [=set of interventions] to ensure appropriate antibiotic use)	yes

**I9**	How many full-time equivalent staff (physician, pharmacist, nurse) are part of the antimicrobial stewardship team and running these stewardship activities on a daily basis in your hospital as part of a dedicated antimicrobial stewardship programme?	2 (yes)
**I10**	Is there a physician identified as a leader for antimicrobial stewardship activities at your facility?	yes
**I11**	Is there a pharmacist responsible for ensuring appropriate antibiotic use at your facility?	yes
**I12**	Does your facility provide any salary support for dedicated time for antimicrobial stewardship activities? (eg, percentage of full-time equivalent staff for ensuring appropriate antibiotic use)	no
**I13**	Does your facility have the information technology (IT) capability to support the needs of the antimicrobial stewardship activities?	yes
**I14**	Does your facility have an outpatient parenteral antibiotic therapy (OPAT) unit?	no
	**Policy and Practice**	
**P1**	Does your facility have an antibiotic formulary (including unrestricted and restricted antibiotics) updated continuously?	no
**P2**	Is your antibiotic formulary based on Essential Drug List?	no
**P3**	Does your facility have an antibiotic guideline?	yes
**P4**	Does your facility have a local antibiotic guideline?	no
**P5**	Are your local antibiotic guidelines based on local antibiotic susceptibility to assist with antibiotic selection for common clinical conditions?	no

**P6**	Does your facility have a written policy that requires prescribers to document an indication in the medical record or during order entry for all antibiotic prescriptions?	no
**P7**	Is it routine practice for specified antibiotic agents to be approved by a physician or pharmacist in your facility? (eg, pre-authorization)	no
**P8**	Is there a formal procedure for a physician, pharmacist, or other staff member to review the appropriateness of an antibiotic at or after 48 hours from the initial order (post-prescription review)?	no
	**Monitoring and Feedback**	
**M1**	Does your facility monitor whether the indication is captured in the medical record for all antibiotic prescriptions?	no
**M2**	Does your facility audit or review surgical antibiotic prophylaxis choice and duration?	no
**M3**	Are results of antibiotic audits or reviews communicated directly with prescribers?	no
**M4**	Does your facility monitor antibiotic use?	yes
**M5**	Does your facility monitor antibiotic use defined by grams (Defined Daily Dose [DDD] or counts (Days of Therapy [DOT]) of antibiotic(s) by patient per day?	no
**M6**	Is monitored antibiotic use reported by hospital activity denominator (by number of admissions/discharges or by number bed-days/patient days)?	no
**M7**	Has an annual report focused on antimicrobial stewardship (summary antibiotic use and/or practices improvement initiatives) been produced for your facility in the past year?	no

**M8**	Has your facility produced a cumulative antibiotic susceptibility report in the past year?	no
**M9**	Is your facility participating in a national antibiotic resistance surveillance programme?	no
**M10**	Is your facility participating in a national antibiotic use surveillance programme?	no
**M11**	How many blood cultures have been made in the past year?	NA
**M12**	List antibiotics out of stock during the survey period	NA
